# The Onset of Double Diffusive Convection in a Viscoelastic Fluid-Saturated Porous Layer with Non-Equilibrium Model

**DOI:** 10.1371/journal.pone.0079956

**Published:** 2013-11-28

**Authors:** Zhixin Yang, Shaowei Wang, Moli Zhao, Shucai Li, Qiangyong Zhang

**Affiliations:** 1 Department of Engineering Mechanics, School of Civil Engineering, Shandong University, Jinan, P.R. China; 2 Geotechnical and Structural Engineering Research Center, Shandong University, Jinan, P.R. China; Plymouth University, United Kingdom

## Abstract

The onset of double diffusive convection in a viscoelastic fluid-saturated porous layer is studied when the fluid and solid phase are not in local thermal equilibrium. The modified Darcy model is used for the momentum equation and a two-field model is used for energy equation each representing the fluid and solid phases separately. The effect of thermal non-equilibrium on the onset of double diffusive convection is discussed. The critical Rayleigh number and the corresponding wave number for the exchange of stability and over-stability are obtained, and the onset criterion for stationary and oscillatory convection is derived analytically and discussed numerically.

## Introduction

The problem of double diffusive convection in porous media has attracted considerable interest during the past few decades because of its wide range of applications, including the disposal of the waste material, high quality crystal production, liquid gas storage and others.

Early studies on the phenomena of double diffusive convection in porous media are mainly concerned with problem of convective instability in a horizontal layer heated and salted from below. The double-diffusive convection instabilities in a horizontal porous layer was studied primarily by Nield [1], [2] on the basis of linear stability theory for various thermal and solutal boundary conditions. Then the analysis is extended by Taunton [3] et al., Turner [4]–[6], Huppert and Turner [7]. Platten and Legros [8] reported excellent reviews about these studies, using subject of extensive theoretical and experimental investigations. Recently, Pritchard and Richardson [9] discussed how the dissolution or precipitation of the solute effect the onset of convection.

On the other hand, viscoelastic fluid flow in porous media is of interest for many engineering fields. Unfortunately, the convective instability problem for a binary viscoelastic fluid in the porous media has not been given much attention. Wang and Tan [10], [11] performed the stability analysis of double diffusive convection of Maxwell fluid in a porous medium, and they pointed out that the relaxation time of Maxwell fluid enhances the instability of the system. Double-diffusive convection of Oldroyd-B fluid in the porous media is studied by Malashetty and co-workers [12]–[14].

In present research, we perform the linear stability of double diffusive convection in a viscoelastic fluid-saturated porous layer, with the assumption that the fluid and solid phases are not in local thermal equilibrium (LTE). The effects of parameters of the system on the onset of convection are discussed analytically and numerically. The critical Rayleigh number, wave number and frequency for exchange of stability are determined.

## Mathematical Model

### Basic Equations

We consider an infinite horizontal porous layer of depth 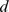, saturated with a Maxwell fluid mixture heated and salted from below, with the vertically downward gravity force 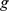 acting on it. The lower surface is held at a temperature 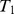 and concentration 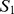, the upper one is kept at a lower temperature 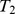 and concentration 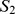. Moreover, 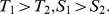


Assuming slow flows in porous media, the momentum balance equation can be linearized as
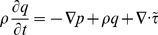
(1)where 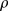 is the density, 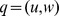 is the volume average velocity obtained by using a volume averaging technique and 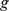 is the acceleration due to gravity, 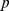 is the pressure.

For general viscoelastic fluids, the constitutive relations between stress tensor 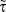 and strain tensor 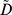 is given by Delenda et al [15]

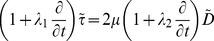
(2)where 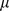 is the viscosity, 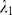 and 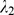 are relaxation time and retardation time, respectively. When the viscoelastic fluid is Maxwell model, 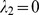. Substituting Eq.(2) into (1), then we get the modified Darcy-Maxwell model to describe the flow in the porous media, neglecting the Soret and Dufour effects between temperature 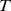 and concentration 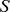 [11], [16]



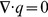
(3)

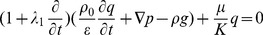
(4)

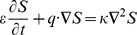
(5)where 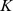 and 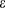 are the permeability and porosity of the medium while 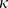 is the effective solutal diffusivity of the medium.

We assume that the diffusion of temperature obeys the following equations, which is a non-equilibrium model between the solid and fluid phases, suggested by [2], [14], [17]

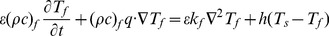
(6)

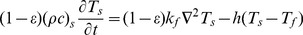
(7)where 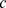 is the specific heat, 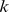 is the thermal conductivity with the subscripts 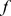 and 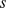 denoting fluid and solid phase respectively, 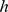 is the inter-phase heat transfer coefficient. The inter-phase heat transfer coefficient 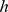 depends on the nature of the porous matrix and the saturating fluid, and the small values of 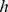 gives rise the relatively strong thermal non-equilibrium effects. In Eqs.(6)–(7), 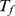 and 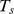 are intrinsic average of the temperature fields and this allows one to set 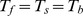, whenever the boundary of the porous medium is maintained at the temperature 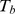.

The onset of double diffusive convection can be studied under the Boussinesq approximation and an assumption that the fluid 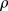 depends linearly on the temperature T and solute concentration S
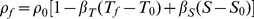
(8)where 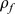 and 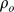 are the densities at the current and reference state, respectively. The quantities 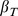 and 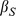 are the coefficients for thermal and solute expansion, respectively. Because of the Boussinesq approximation, which states that the effect of compressibility is negligible everywhere in the conservations except in the buoyancy term, is assumed to hold.

### Basic State

The basic state is assumed to be quiescent and we superimpose a small perturbation on it. We eliminate the pressure from the momentum transport equation(4) and define stream function 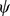 by
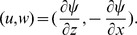



Then the following dimensionless variables are defined as
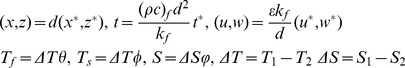
(9)


Here the symbol “
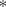
” means dimensionless, and 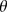, 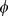 are non-dimensional temperatures of fluid and solid phase, respectively. 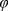 is non-dimensional concentration of solute in porous medium. Substituting the above dimensionless variables in the system yields the following non-dimensional governing equations (for simplicity, the dimensionless mark “*” will be neglected hereinafter)
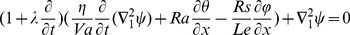
(10)

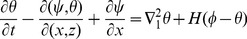
(11)

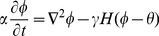
(12)

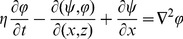
(13)where 
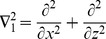
 is the two-dimensional Laplacian operator, and the non-dimensional variables that appear in the above equations are defined as
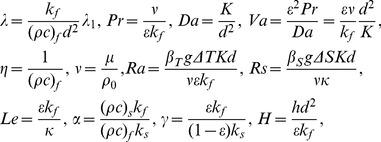
(14)where the 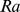 is the thermal Rayleigh number, 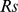 is the solute Rayleigh number, 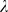 is the relaxation parameter, 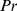 is the Prandtl number, 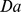 is the Darcy number, 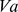 is the Vadasz number, 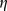 is the normalized porosity, 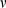 is the kinematic viscosity, 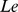 is the Lewis number, 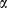 is the diffusive ratio, 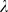 is the porosity modified conductivity ratio, 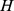 is the non-dimensional interphase heat transfer coefficient. When 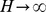, the solid and fluid phase have almost equal temperatures; and for small values of 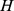, the solid phase ceases to affect the thermal field of the fluid.

Hence the boundary conditions are
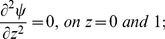


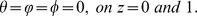



### Linear Stability Theory

In this section, we discuss the linear stability of the system. According to the normal mode analysis, the Eqs.(10)–(13) is solved using the time dependent periodic disturbances in a horizontal plane. We assume that the amplitudes are small enough, so the perturbed quantities can be expressed as follows
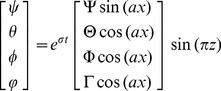
(15)


Where 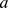 is the horizontal wavenumber, and 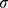 is the growth rate. Substitution of Eq.(15) into the linearized version of Eqs.(10)–(13), yields the following equation:
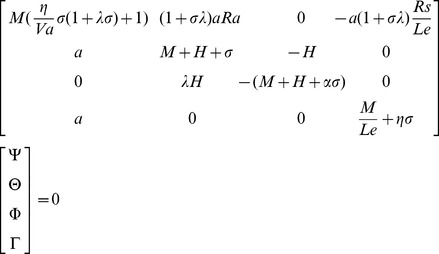
(16)where 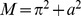. For non-trivial solution, the determinant of the coefficient matrix must be zero. Therefore, by setting the determinant of the coefficient matrix to zero we get



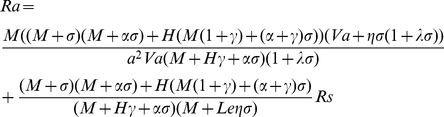
(17)The growth rate 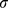 is in general a complex quantity such that 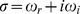. The system with 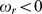 is always stable, while for 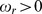, it will unstable. For the neutral stability state 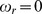, we set 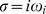 in the Eq.(17) and clear the complex quantities from the denominator, to obtain



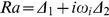
(18)where



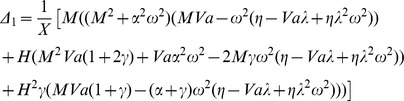


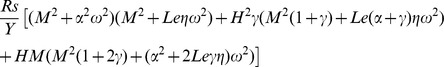


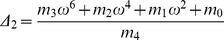


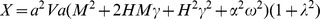


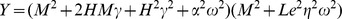


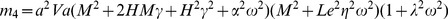


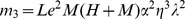


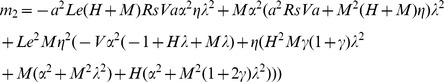


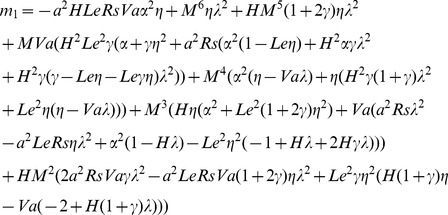


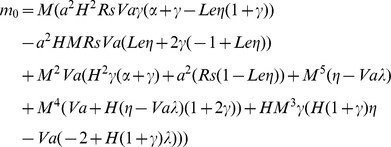



Since 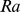 is a physical quantity, it must be real. Hence, from Eq.(18) it follows that either 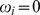 (steady onset) or 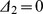 (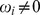, oscillatory onset).

### Stationary Convection

The steady onset corresponds to 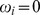 and reduces the Eq.(18) to
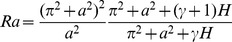
(19)


This result is obtained by Banu and Rees [18] in the case of a Darcy porous medium with thermal non-equilibrium model. When 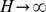, in the case of local thermal equilibrium Eq.(17) takes the form
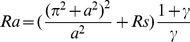
(20)


Further Eq.(20) can be written as
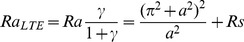
(21)


In the absence of the solute effect, Eq.(21) reduces to
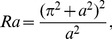
(22)which is the classical result, obtained by Horton and Rogers [19].

The value of Rayleigh number 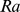 given by Eq.(17) can be minimized with respect to the wavenumber a by setting 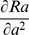 and solve the equation
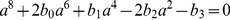
(23)

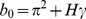


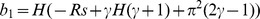


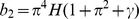


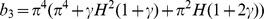



### Asymptotic Analysis

Case 1: For very small values of 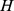.

When the value of H is very small, the critical value of the Rayleigh number 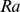 is slightly above the critical value for the LTE case. We expand 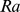 given by Eq.(17) in a power series in H as
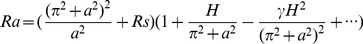
(24)


To minimize 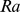 up to 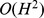, we set 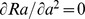 and we obtain an expression of the form
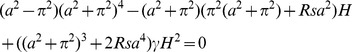
(25)


We also expand 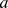 in power series of 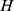 as



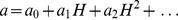
(26)where 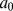 is the critical wavenumber for the LTE case,we obtain 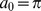 from the Eq.(21).

Substituting Eq.(26) into the Eq.(25), and rearranging the terms and then equating the coefficients of same powers of H will allow us to obtain the 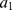 and 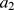, we get
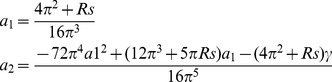
(27)


Substituting these values of 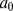, 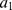 and 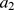 into the Eq.(25), we can obtain the critical Rayleigh number for small H.

Case 2: For very large values of 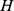.

For the very large values of 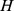, the critical Rayleigh number expand in a power series with respect to 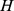 as
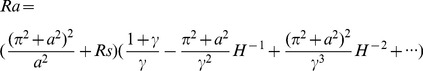
(28)


Letting 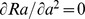, we obtain the following expression
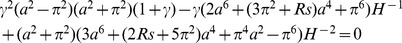
(29)


Similarly, we expand 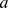 in power series of 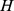 as
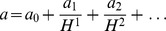
(30)


Substituting Eq.(30) into the Eq.(29), we get
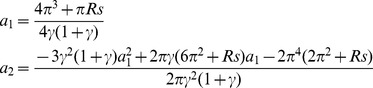
(31)


Then, substituting these values of 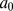, 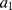 and 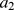 into the Eq.(28), we can obtain the critical Rayleigh number for large 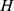.

### Oscillatory Convection

For oscillatory onset 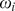 is non-zero, which requires 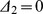 in (18), giving
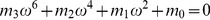
(32)which has be rewritten as a dispersion relation which is cubic in 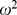. Now Eq.(18) with 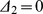, obtain



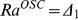
(33)
Equation (33) implies that for some wavenumber, there may exist more than one real positive values of 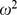, corresponding to different onset frequencies for that value of 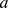. To each such frequency there corresponds a Rayleigh number 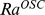 on the oscillatory neutral curve. Moreover, it note that we cannot have two different frequencies at the same wavenumber 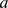. That is, there can be at most one of these stratifying agencies will be unstabilizing. To find the extremal value(s) of oscillatory Rayleigh number, we first determine the number of positive solutions of Eq.(32). If there are none, then no oscillatory instability is possible. If there are more than one, then the minimum of Eq.(33) with 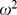 obtained by Eq.(32) gives the oscillatory neutral Rayleigh number.The analytical expression for oscillatory Rayleigh number given by Eq.(33) is minimized with respect to the wavenumber numerically, after substituting for 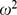 from Eq.(32), for various values of physical parameters in order to know their effects on the onset of oscillatory convection.

### Numerical Results and Discussion

**Figure 1:**
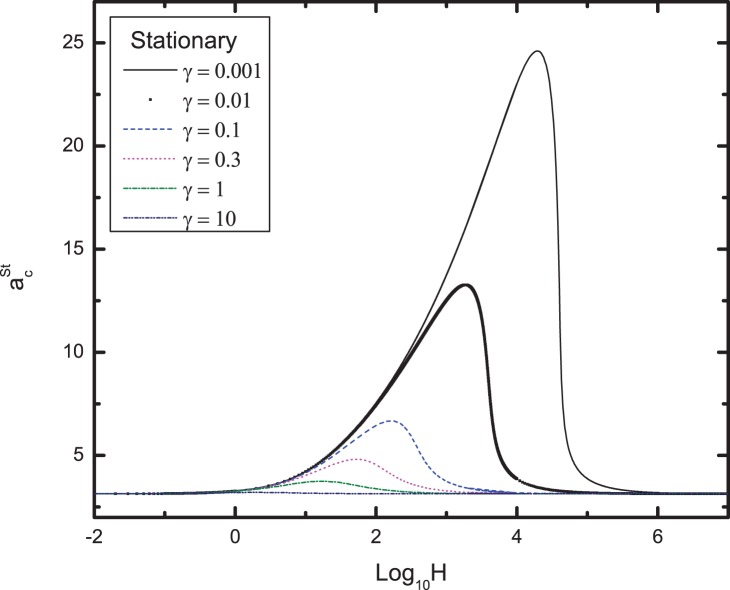
Variation of the critical wavenumber 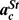 for stationary mode with H for different values of 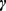.

Figure 1 illustrates the variation of the critical wavenumber for stationary mode 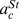 with 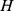 for different values of the conductivity ratio 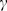. It can be seen from the figure that as the value of 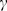 increases from 0.001 to 10, the critical wavenumber 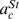 decreases. On the other hand, the value of wavenumber 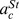 approaches a common limits and becomes independent of the 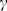 when the 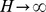 and 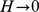.

**Figure 2:**
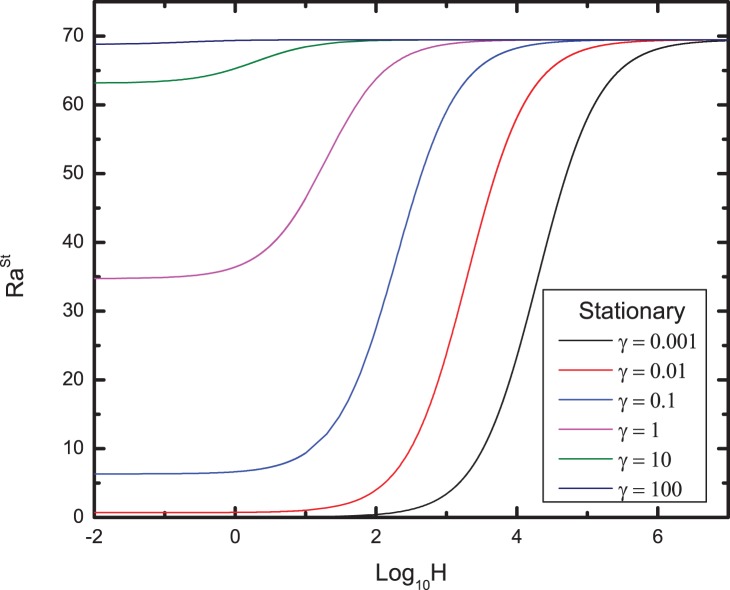
Variation of the critical Rayleigh number for stationary mode based on the mean properties of the porous medium with H for different values of conductivity ratio 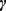.

Effect of different values of conductivity ratio 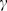 on the Rayleigh number profiles 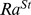 for stationary mode are shown in Fig. 2. An increase in 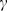 leads to a decrease in 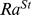, which means the increasing 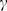 stabilizes the system. Moreover, when 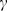 becomes very large, the effect of 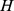 on 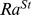 can be neglected, and the effect of 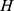 is negligible at small value of 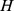. For the intermediate of 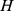, the critical Rayleigh number 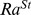 increases with increasing values of 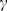.

**Figure 3:**
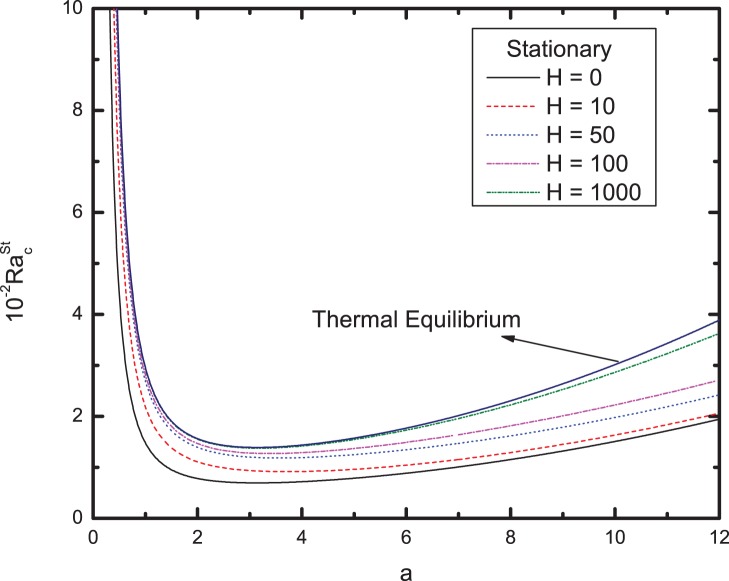
Variation of the critical Rayleigh number for stationary mode with wavenumber a for different values of the heat transfer coefficient H.

**Figure 4:**
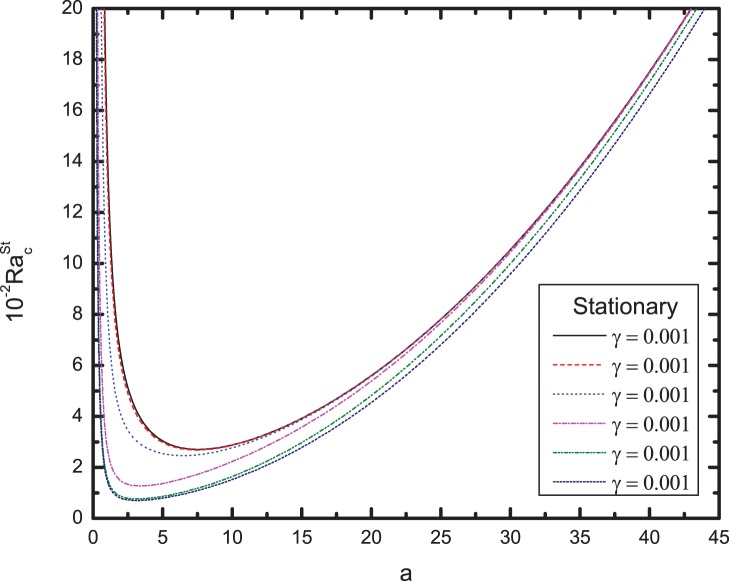
Variation of the critical Rayleigh number for stationary mode with wavenumber 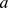 for different values of conductivity ratio 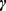.

Figures 3 and 4 summarize these results, which show the effects of varying values of 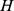 and 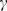 on the critical Rayleigh number for stationary mode 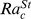, respectively. It is quite clear from Fig. 3 that the value of 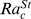 for any chosen wavenumber increases with increasing the value of heat transfer coefficient 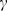. Furthermore, there exists a corresponding shift in the position of the minimum peak in Fig. 3 and 4. Moreover, the larger the heat transfer coefficient 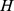 is, the faster the heat transfer enabling the viscoelastic fluid to attain greater percolation velocity. Therefore large heat transfer coefficient favors onset of convection. From Figs. 4, we observe that the effect of increasing 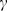 decreases the minimum of the Rayleigh number for stationary mode, indicating that the effect of the porosity modified conductivity ratio is to advance the onset of convection.

**Figure 5:**
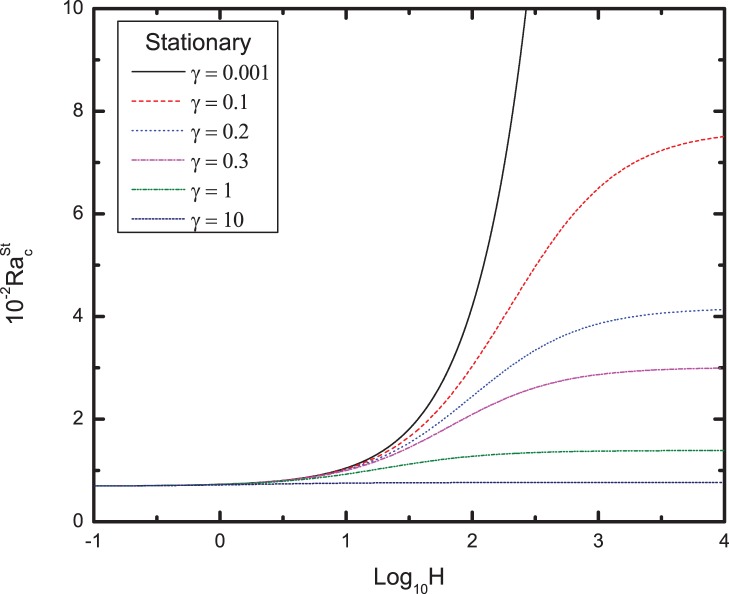
Variation of the critical Rayleigh number for stationary mode with the heat transfer coefficient 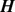 for different values of conductivity ratio 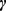.

The variation of conductivity ratio on the critical Rayleigh number for stationary mode with the heat transfer coefficient for different values of conductivity ratio is shown in Fig. 5. We find that the critical Rayleigh number is independent of 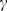 for small values of 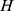, but for large 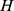, the critical Rayleigh number decreases with increasing 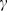. Moreover, for very large 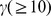, the critical Rayleigh number is independent of 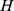. Thus, we can draw the conclusion that the presence of non-equilibrium of heat transfer between the viscoelastic fluid and solid make the system instable.

**Figure 6:**
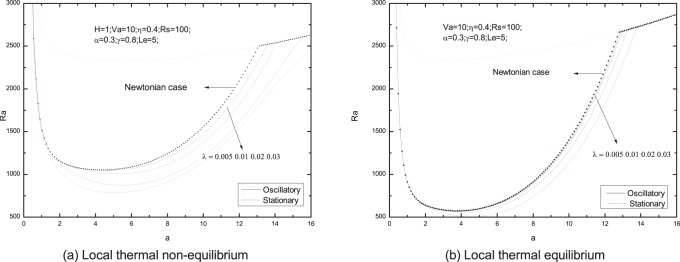
Neutral stability curves for different values of stress relaxation time 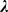.

**Figure 7:**
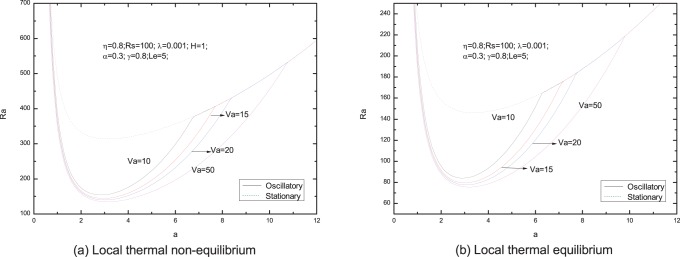
Neutral stability curves for different values of Vadasz number 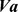.

**Figure 8:**
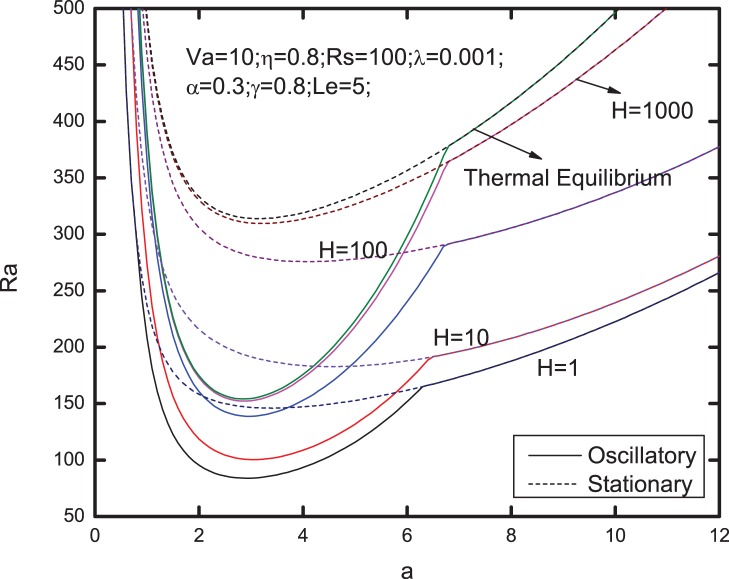
Neutral stability curves for different values of heat transfer coefficient 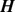.

**Figure 9:**
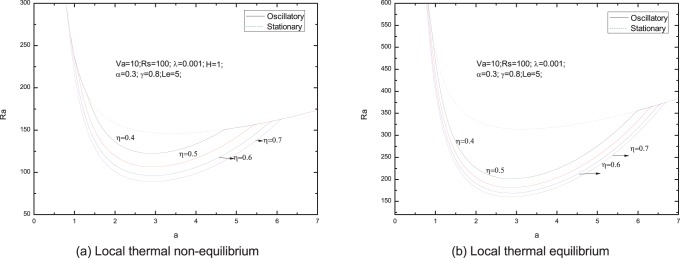
Neutral stability curves for different values of normalized porosity parameter 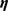.

**Figure 10:**
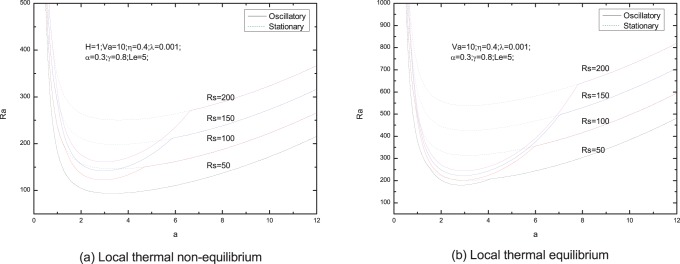
Neutral stability curves for different values of solute Rayleigh number 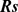.

**Figure 11:**
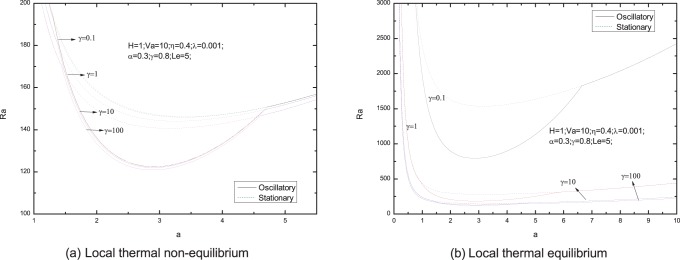
Neutral stability curves for different values of porosity modified conductivity ratio 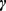.

**Figure 12:**
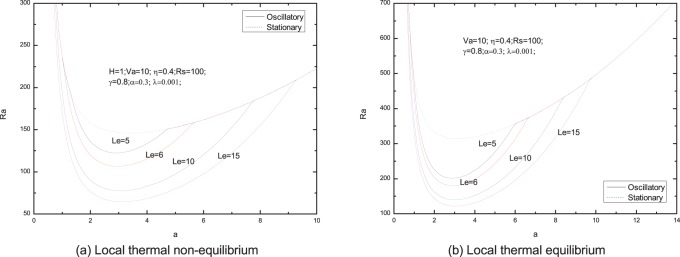
Neutral stability curves for different values of Lewis number 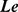.

**Figure 13:**
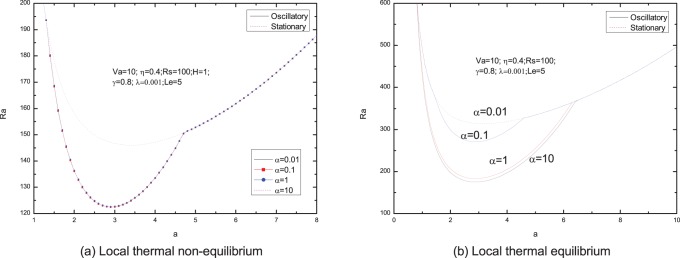
Neutral stability curves for different values of diffusivity ratio 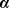.

Fig. 6–13 present the neutral curves for different values of the relaxation parameter 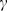, Vadasz number, heat transfer coefficient 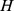, normalized porosity parameter 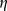, solute Rayleigh number 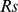, porosity modified conductivity ratio 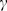, Lewis number 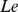 and diffusivity ratio 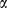, respectively. As can be seen from the figures, these parameters has significant effects upon the neutral curves.

The effect of relaxation time on the neutral curves is shown in Fig. 6. It is shown in Fig. 6a, i.e., for local thermal non-equilibrium case, the minimum of the Rayleigh number is smaller when 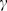 is larger, which makes the onset of convection easier. Based on the theory of Maxwell fluid model, a fluid relaxation or characteristic time, 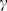, is defined to quantify the viscoelastic behavior [20]. So we draw a conclusion that the physical mechanism is the increasing relaxation time increases the elasticity of a viscoelastic fluid thus causing instability. As a result, the elasticity of the Maxwell fluid has a destabilizing effect on the fluid layer in the porous media, and the oscillatory convection is easy to occur for viscoelastic fluid. And this result agrees with the result given by Wang and Tan [11], where they studied the double diffusive convection problem with thermal equilibrium, as shown in Fig. 6b.

From Fig. 7, We find that an increase in the value of the Vadasz number decreases the oscillatory Rayleigh number, indicating that the Vadasz number advances the onset of double diffusive convection, which is in agreement with the literature by Malashetty and Biradar [16].

The stationary Rayleigh number increases with an increase in the value of heat transfer coefficient 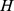, as shown in Fig. 8, indicating that the effect of heat transfer coefficient is to enhance the stability of the system. At the same time, the same effect of 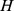 upon the oscillatory Rayleigh number can be observed in this figure. Comparing with the curve for local thermal equilibrium model, it can be seen that the the oscillatory convection is easy to occur for thermal non-equilibrium case.

In Fig. 9, we note that the effect of normalized porosity parameter is to advance the onset of oscillatory convection. From Fig. 10, we find that the increasing 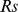 has a stabilizing effect on the onset of double diffusive convection. The neutral stability curves for stationary and oscillatory modes for different values of porosity modified conductivity ratio is shown in Fig. 11, which leads us to the conclusion that the increasing porosity modified conductivity ratio has a destabilizing effect for the system.

The effect of Lewis number 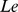 on the critical oscillatory Rayleigh number is shown in Fig. 12. From the figure, it can be found that increasing of Lewis number decreases the critical oscillatory Rayleigh number indicating that the Lewis number destabilizes the system in oscillatory mode. The physical interpretation has been given by Malashetty and Biradar [16], when 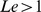, the diffusivity of heat is more than that of solute, and therefore, destabilizing solute gradient augments the onset of oscillatory convection. From Fig. 13, we observe that the diffusivity ratio 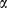 has little effect on the onset of double diffusive convection.

## Conclusion

The onset of double diffusive convection in a binary Maxwell fluid, which is heated and salted from below, is studied analytically using using a thermal non-equilibrium model. Based on the normal mode technique, the linear stability has been studied analytically. The effects of relaxation time, heat transfer coefficient, normalized porosity parameter and other parameters on the stationary and oscillatory convection are discussed and shown graphically. It is found that the increasing relaxation time increases the elasticity of a viscoelastic fluid thus causing instability. The asymptotic solutions for both small and large values of 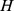 were obtained. In general, this work showed how the relaxation time and non-equilibrium model affects the double-diffusive convection in porous media, and it may be useful in some applications which contains heat and mass transfer.

## References

[1] Nield D (1968) Onset of thermohaline convection in a porous medium. Water Resour. Res. 4: 553–560.

[2] Nield D, Bejan A (2006) Convection in Porous Media, 3rd ed., New York: Springer.

[3] Taunton J, Lightfoot E, Green T (1972) Thermohaline instability and salt fingers in a porous medium. Phys. Fluids 15: 748–753.

[4] Turner J (1973) Buoyancy Effects in Fluids. London: Cambridge University Press.

[5] Turner J (1974) Double diffusive phenomena. Ann. Rev. Fluid Mech. 6: 37–56.

[6] Turner J (1985) Multicomponent convection. Ann. Rev. Fluid Mech. 17: 11–44.

[7] Huppert H, Turner J (1981) Double diffusive convection. J. Fluid Mech. 106: 299–329.

[8] Platten J, Legros J (1984) Convection in Liquids. Berlin: Springer.

[9] Pritchard D, Richardson C (2007) The effect of temperature-dependent solubility on the onset of thermosolutal convection in a horizontal porous layer. J. Fluid Mech. 571: 59–95.

[10] Wang S, Tan W (2008) Stability analysis of double-diffusive convection of Maxwell fluid in a porous medium heated from below. Phys. Lett. A 372: 3046–3050.

[11] Wang S, Tan W (2011) Stability analysis of soret-driven double-diffusive convection of Maxwell fluid in aporous medium. Int. J. Heat Fluid Flow 32: 88–94.

[12] Malashetty M, Shivakumara I, Kulkarni S (2005) The onset of Lapwood-Brinkman convection using a thermal non-equilibrium model. Int. J. Heat Mass Tran. 48: 1155C1163.

[13] Malashetty M, Shivakumara I, Kulkarni S (2009) The onset of convection in a couple stress fluid saturated porous layer using a thermal non-equilibrium model. Phys. Lett. A 373: 781–790.

[14] Malashetty M, Hill A, Swamy M (2012) Double diffusive convection in a viscoelastic fluid-saturated porous layer using a thermal non-equilibrium model. Acta Mech. 223: 967–983.

[15] Delenda N, Hirata S, Ouarzazi M (2012) Primary and secondary instabilities of viscoelastic mixtures saturating a porous medium: Application to separation of species. J. Non-Newton. Fluid. Mech. 181C182: 11C21.

[16] Malashetty M, Biradar S (2011) The onset of double diffusive convection in a binary Maxwell fluid saturated porous layer with cross-diffusion effects. Phys. Fluids 23: 1–13.

[17] Chen X, Wang S, Tan W (2011) Stability analysis of thermosolutal convection in a horizontal porous layer using a thermal non-equilibrium model. Int. J. Heat Fluid Flow 32: 78–87.

[18] Banu N, Rees D (2002) Onset of Darcy-Benard convection using a thermal non-equilibrium model. Int. J. Heat Mass Tran. 45: 2221C2228.

[19] Horton C, Rogers F (1945) Convection currents in a porous medium. Journal of Applied Physics 16: 367–370.

[20] Chhabra R, Richardson J (1999) Non-Newtonian Flow in the Process Industries. London: Butterworth-Heinemann.

